# Linkage studies of catechol-*O*-methyltransferase (*COMT*) and dopamine-beta-hydroxylase (*DBH*) cDNA expression levels

**DOI:** 10.1186/1753-6561-1-s1-s95

**Published:** 2007-12-18

**Authors:** Chao Xing, Monica Torres-Caban, Tao Wang, Qing Lu, Guan Xing, Robert C Elston

**Affiliations:** 1 Department of Epidemiology and Biostatistics, Case Western Reserve University, Wolstein Research Building 2103, Cornell Drive, Cleveland, Ohio 44106, USA; 2 Department of Clinical Sciences, University of Texas Southwestern Medical Center, 5323 Harry Hines Boulevard, Dallas, Texas 75390-8591, USA; 3 McDermott Center for Human Growth and Development, University of Texas Southwestern Medical Center, 5323 Harry Hines Boulevard, Dallas, Texas 75390-8591, USA

## Abstract

The *COMT *and *DBH *genes are physically located at chromosomes 22q11 and 9q34, respectively, and both *COMT *and *DBH *are involved in catecholamine metabolism and are strong candidates for certain psychiatric and neurological disorders. Although the genetic determinants for both enzymes' activities have been widely studied, their genetic involvement on gene mRNA expression levels remains unclear. In this study we performed quantitative linkage analysis of *COMT *and *DBH *cDNA expression levels, identifying transcriptional regulatory regions for both genes. Multiple Haseman-Elston regression was used to detect both additive and interactive effects between two loci. We found that the master transcriptional regulatory region 20q13 had an additive effect on the *COMT *expression level. We also found that chromosome 19p13 showed both additive and interactive effects with 9q34 on *DBH *expression level. Furthermore, a potential interaction between *COMT *and *DBH *was indicated.

## Background

Catechol-*O*-methyltransferase (*COMT*) (MIM 116790) catalyzes the transfer of a methyl group from S-adenosyl-methionine to catecholamines [1], and dopamine-beta-hydroxylase (*DBH*) (MIM 223360) catalyzes the conversion of dopamine to norepinephrine [2]; both enzymes are involved in the catecholamine metabolism system, in which abnormalities are hypothesized to be related to the development of psychiatric and neurological disorders such as schizophrenia and depression (for reviews, see [3,4]). There are two distinct forms of COMT, a membrane-bound COMT (MB-COMT) and a soluble COMT (S-COMT) found in the cell cytoplasm [5]; the former is predominantly expressed in brain neurons, whereas the latter is predominantly expressed in other tissues such as liver and kidney. DBH is expressed in the vesicles of central noradrenergic and adrenergic neurons, peripheral noradrenergic neurons, and adrenomedullary neuronsecreotry cells [6]. The *COMT *and *DBH *genes are physically located at chromosomes 22q11 and 9q34, respectively [7,8].

There has been intensive research on the genetic determinants of plasma COMT and DBH activities, and findings so far strongly suggest that the structural genes *COMT *and *DBH *encoding these proteins are the major quantitative trait loci for their respective plasma activities; moreover, in both genes single-nucleotide polymorphisms (SNPs) have been identified that may account for most of the variation in the enzyme activity levels. On the common functional SNP rs165688, a G→A substitution in exon 4 of *COMT *produces a valine→methionine (Val/Met) substitution at codons 158 and 108 in the MB-COMT and S-COMT transcripts, respectively. This polymorphism has been shown to have a functional effect on enzyme activity; in particular, Val is a predominant factor that determines higher COMT activity [9]. The SNP rs1611115 (-1021C→T) located in the 5' flanking region of the *DBH *gene accounts for 35% to 52% of the variance in plasma DBH activity in populations from diverse geographic origins [10].

Though the major effects of these common variants on enzyme activities have been replicated in independent samples, their effects on gene mRNA expression levels remain unclear. The effect of SNP rs165688 on *COMT *mRNA expression level has been controversial. Bray et al. [11] showed that it altered the *COMT *mRNA expression level; in contrast, several other studies failed to detect this association between *COMT *genotypes and expression level (for examples, see [12]). So far, studies on the determinants of *COMT *expression level have been limited to seeking *cis*-acting regulators, including SNP rs165688 and several other SNPs within *COMT*. To our knowledge, there is as yet no literature documenting effects of SNPs on *DBH *mRNA expression levels.

Phenotypic variation in the transcript level represents an intermediate stage between DNA sequence differences and the clinical expression of complex traits, and there have been studies suggesting a genetic contribution to the variation in gene expression level [13-15]. In the current study, we performed quantitative linkage analysis for both *COMT *and *DBH *expression phenotypes, identifying transcriptional regulatory regions for both genes. For *COMT*, we first identified *cis*-acting influences on the gene expression level, and then we performed a genome-wide linkage scan for *trans*-acting regulators across the genome conditional on the *cis*-acting effect at chromosome 22q11. For *DBH*, we similarly first identified the *cis*-acting influences on the gene expression level, and then, both conditional and unconditional on the *cis*-acting effect at chromosome 9q34, performed a linkage scan for *trans*-acting regulators specifically on chromosome 19, based on the evidence from previous linkage studies [16-18] that there might be a second locus near chromosome 19p13 acting jointly with *DBH *at chromosome 9q34 to influence DBH plasma activity.

## Methods

The study subjects consisted of 14 three-generation CEPH (Centre d'Etude du Polymorphisme Humain) Utah pedigrees, including 371 full sibling pairs. 2,820 SNPs were relatively evenly genotyped across the 22 autosomes by the SNP Consortium [19]. Treating the expression level of *COMT *and *DBH *in lymphoblastoid cells of the above subjects as a quantitative trait, we applied Haseman-Elston regression model-free linkage analyses [20,21], as implemented in the SIBPAL program. In particular, only data on sib pairs were considered (thus avoiding the complication of possible intergenerational heterogeneity) and the generalized estimating equation technique [22] was utilized to allow for non-independence of sib pairs within a sibship. The basic Haseman-Elston (HE) regression can be written in the form

$$Y=\beta_{0}+\beta_{1}{\hat{\pi }}_{i},$$

where *Y *is a function of traits for a sib pair, ${\hat{\pi }}_{i}$ is the estimated proportion of alleles shared identically by descent (IBD) at marker locus *i *between members of a sib pair, *β*_0 _is the intercept, and *β*_1_, the slope, is the locus-specific genetic variance of the trait attenuated by recombination between the trait and marker loci. To detect *trans*-acting effects at other genomic regions conditional on the *cis*-acting effect at the structural gene loci (22q11 and 9q34 for *COMT *and *DBH*, respectively), we performed the multiple HE regression

$$Y=\beta_{0}+\beta_{1}{\hat{\pi }}_{0}+\lambda {\hat{\pi }}_{1},$$

where the subscript 0 denotes a marker for the structural gene locus and the subscript 1 denotes another locus in the genome. The coefficient *λ *denotes the (attenuated) genetic variance at locus 1 and a value significantly different from zero indicates an additive effect of locus 1 with locus 0 on gene expression level. We further investigated genetic interaction between the structural gene locus and the other locus with the model

$$Y=\beta_{0}+\beta_{1}{\hat{\pi }}_{0}+\beta_{2}{\hat{\pi }}_{1}+\gamma {\hat{\pi }}_{0}{\hat{\pi }}_{1},$$

where *γ *denotes variance (again, attenuated by recombination) due to the genetic interaction between locus 1 and locus 0, and a value significantly different from zero indicates an interactive effect of the two loci on gene expression level. Single-point IBD sharing was estimated by the program GENIBD. Both of the above programs are included in the Statistical Analysis for Genetic Epidemiology (S.A.G.E.) software [23].

## Results

### Linkage of *COMT *expression levels

We detected a linkage signal at chromosome 22q11 (*p *= 6.5 × 10^-3^). The closest SNP to the peak of the signal is rs738842, which is ~3 megabases (Mb) centrometric to the *COMT *gene and within the *cis*-acting region defined by Morley et al. [15]. There were 10 regions showing only additive *trans*-acting effects (Table 1) and 7 regions showing only interactive *trans*-acting effects (Table 2) at the nominal significance level of 0.005; at 14 other regions, both effects attained a nominal significance level of 0.05 and at least one attained the level 0.005 (Table 3). These nominal significance levels were arbitrarily chosen in order to report the most notable results. Note that 1) chromosome 9q34, where *DBH *is located, showed an additive *trans*-acting effect with 22q11 on *COMT *expression level; and 2) chromosome 20q13, which was one of the two hot spots of transcriptional regulation detected by Morley et al. [15], was also detected to have an additive effect with 22q11 on the *COMT *expression level.

**Table 1:** Chromosome regions showing only additive *trans*-acting effects^a ^with 22q11 on *COMT *expression level

Genomic region	Closest marker	Physical distance (Mb)	*p*-Value for *λ*
3q26	rs1499812	173.2	4.4 × 10^-3^
4q35	rs1979255	190.7	3.4 × 10^-4^
5q34	rs1387395	163.8	4.1 × 10^-3^
6p22	rs1475149	21.8	2.5 × 10^-3^
7p21	rs988418	9.9	3.7 × 10^-3^
8q22	rs1075394	101.5	1.8 × 10^-3^
9q34	rs936249	138.2	2.2 × 10^-3^
10p11	rs1857063	29.4	2.0 × 10^-7^
18p11	rs321663	8.6	2.1 × 10^-4^
20q13	rs756529	47.4	3.1 × 10^-3^

^a^At a nominal level of 0.005

**Table 2:** Chromosome regions showing only interactive *trans*-acting effects^a ^with 22q11 on *COMT *expression level

Genomic region	Closest marker	Physical distance (Mb)	*p*-Value for *γ*
1q32	rs871446	196.9	1.9 × 10^-3^
3p26	rs1385466	4.0	1.2 × 10^-5^
6q23	rs2040034	137.3	3.9 × 10^-4^
9q33	rs1326808	116.7	2.1 × 10^-3^
10q22	rs1907308	78.0	8.3 × 10^-6^
15q26	rs288423	96.0	3.3 × 10^-3^
18q21	rs1943985	45.5	1.5 × 10^-4^

^a^At a nominal level of 0.005

**Table 3:** Chromosome regions showing both additive and interactive *trans*-acting effects^a ^with 22q11 on *COMT *expression level

			*p*-Value	
			
Genomic region	Closest marker	Physical distance (Mb)	*λ*	*γ*
1q41	rs1561189	213.5	1.1 × 10^-4^	4.6 × 10^-3^
2q35	rs1425118	216.8	<1.0 × 10^-8^	3.5 × 10^-2^
3q28	rs1559018	191.8	2.3 × 10^-2^	2.9 × 10^-5^
4q31	rs795995	141.1	1.5 × 10^-3^	5.0 × 10^-7^
5q14	rs1020720	80.5	1.7 × 10^-2^	1.8 × 10^-3^
6q16	rs2021678	102.1	1.1 × 10^-3^	4.7 × 10^-2^
7p14	rs2051936	40.8	5.7 × 10^-3^	3.5 × 10^-3^
8p23	rs433960	12.9	4.7 × 10^-2^	2.9 × 10^-5^
9p23	rs1412307	13.0	1.3 × 10^-4^	6.6 × 10^-3^
14q13	rs1983667	36.2	1.4 × 10^-4^	1.1 × 10^-2^
16p13	rs1981492	0.3	1.0 × 10^-3^	5.9 × 10^-3^
16q24	rs880275	84.8	4.7 × 10^-3^	2.6 × 10^-3^
17p12	rs433068	15.2	4.8 × 10^-2^	4.4 × 10^-3^
22q13	rs960362	47.4	4.8 × 10^-4^	8.6 × 10^-3^

^a^Both effects attained a nominal significance level of 0.05 and at least one reached the level 0.005.

### Linkage of *DBH *expression levels

**Figure 1:**
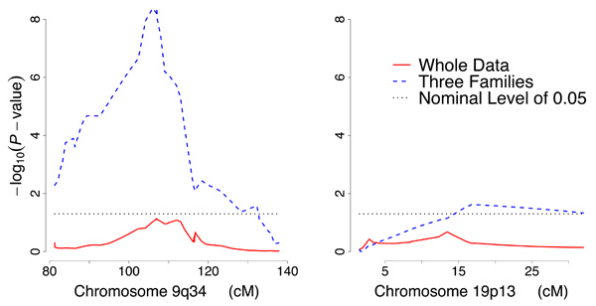
Linkage signals of *DBH *expression levels at chromosomes 9q34 and 19p13.

There was no linkage signal for the *DBH *expression level at either chromosome 9q34 or chromosome 19p13 when analyzing all of the 14 pedigrees together. There could be many reasons for the lack of any linkage signal at chromosome 9q34, the structural gene location (see Discussion). However, upon analyzing the pedigrees individually, it was noticed that three pedigrees (1340, 1345 and 1318), which included 70 full sibling pairs, showed a positive linkage signal at 9q34. Because our primary interest was to test whether there is a second locus near chromosome 19p13 related to *DBH*, in addition to the *DBH *gene, we selected these three pedigrees for further study and found that a linkage signal at 19p13 then also emerged, showing both additive and interactive *trans*-acting effects (*p *= 2.4 × 10^-2 ^and *p *= 2.6 × 10^-2^, respectively) with the locus at chromosome 9q34 on *DBH *expression level (Fig. 1).

## Discussion

Phenotypic variation in the transcript level of a gene involves genetic components; however, the transcriptional regulatory elements may or may not lie in the structural gene itself. Studies by Morley et al. [15] have suggested the existence of master regulatory regions, and we identified chromosome 20q13, nominated by them, as regulating *COMT *expression level. Note that Morley et al. [15] performed simple HE regression, while we performed multiple HE regression so as to condition on the IBD sharing at *COMT*, i.e., our analysis refined their original investigation and provided further evidence supporting chromosome 20q13 as a regulatory region. In the current data, overall there was a linkage signal at 22q11 for *COMT *expression level, but no signal at 9q34 for *DBH *expression level. Lack of an overall linkage signal at 9q34 for *DBH *could be due to there not being enough *DBH *polymorphism segregation in the sample, to there being less expression variation among individuals than between replicates within an individuals [15], from low levels of transcript abundance in view of its tissue-specific expression pattern (Dr. Joseph F. Cubells, personal communication), or to genetic heterogeneity, as also indicated in the earlier study of plasma DBH activity [16].

In this study we employed multiple HE regression to detect additive and interactive effects between two loci. In the three pedigrees selected for having a positive linkage signal at 9q34 for *DBH *expression level, we detected a boost of signal at chromosome 19p13, where previous studies [16-18] indicated the existence of a locus influencing DBH plasma activity. The multiple HE regression indicated both additive and interactive effects of the two loci, suggesting epistasis between them. For simplicity, and because of the limited amount of data available, we did not dissect the total genetic variance into additive and dominance components in this study; with more data the interaction could be characterized by more elaborate models [24], and even embedded in a general pedigree framework [25,26]. We also noticed that chromosome 9q34, where *DBH *is located, showed an additive *trans*-acting effect with 22q11 on *COMT *expression level. This is not totally unexpected because both enzymes are involved in catecholamine metabolism; however, we could not find in the literature any genetic study on the interaction between them, which is surprising. Interaction between *COMT *and *DBH *could be a potential research topic.

In summary, we performed quantitative linkage analysis for both *COMT *and *DBH *expression phenotypes, identifying transcriptional regulatory regions for both genes. Multiple HE regression was used to detect additive and interactive effects between two loci. The master transcriptional regulatory region 20q13 had an additive effect on the *COMT *expression level. Chromosome regions 9q34 and 19p13 acted epistatically on *DBH *expression level. Potential interaction between *COMT *and *DBH *was indicated.

## Competing interests

The author(s) declare that they have no competing interests.
